# Different aspects of impulsivity in chronic alcohol use disorder with and without comorbid problem gambling

**DOI:** 10.1371/journal.pone.0227645

**Published:** 2020-01-30

**Authors:** Ildikó Kovács, Ildikó Demeter, Zoltán Janka, Zsolt Demetrovics, Aniko Maraz, Bálint Andó

**Affiliations:** 1 Department of Psychiatry, Faculty of Medicine, University of Szeged, Szeged, Hungary; 2 Institute of Psychology, Eötvös Loránd University, Budapest, Hungary; 3 Institute für Psychologie, Humboldt-Universität zu Berlin, Berlin, Germany; University of Auckland, NEW ZEALAND

## Abstract

**Background and aims:**

Alcohol use disorder (AUD) and problem gambling are highly comorbid disorders. This study aims to explore the role of four aspects of impulsivity (trait concept of impulsivity, choice impulsivity, impulsive aggression and response inhibition/decision-making) in long-term chronic AUD patients with and without problem or pathological gambling symptoms.

**Methods:**

Cognitively intact chronic AUD patients were enrolled with (n = 32) and without (n = 71) problem gambling symptoms in an inpatient clinic for chronic alcohol users. Multiple facets of impulsivity, cognitive ability, psychopathological symptoms, alcohol and gambling severity were measured.

**Results:**

Chronic AUD patients with gambling disorder symptoms showed longer lifetime alcohol consumption, more severe alcohol use and higher psychopathological symptom severity than AUD patients without gambling symptoms. Gambling severity correlated with overall trait impulsivity, but not with choice impulsivity, impulsive aggression or cognitive impulsivity with controlling for lifetime alcohol consumption, lifetime alcohol use and psychopathological symptom severity. High trait impulsivity and non-planning was associated with comorbid gambling symptoms in AUD patients, which was independent of the level of intelligence, age and psychopathological symptoms.

**Conclusion:**

Comorbid gambling disorder symptoms in chronic AUD was connected to more severe alcohol-related variables. Higher trait impulsivity was also linked with gambling disorder symptoms in patients with chronic AUD. This accents the need of special focus on comorbid GD symptoms in AUD, since prognosis and treatment for them may vary.

## Introduction

Alcohol use disorder (AUD) is one of the most frequently occurring substance use disorder, and it exhibits exceptionally high lifetime prevalence rate (30.3%) [1]. Chronic AUD is highly comorbid with many systemic diseases and is often present simultaneously with other mental disorders [2], from which the high co-occurrence of gambling disorder (GD) and AUD is well established. Studies have shown that AUD and GD are highly comorbid both in community [3,4] and in treatment seeking samples [5,6]. In a nationally representative US study, almost three-quarters (73.2%) of lifetime GD patients had AUD [7], while a population-based meta-analysis reported that 28% of lifetime GD patients report AUD as well [8], and other studies reported a 17–33% comorbidity of current GD in AUD patients [9,10]. AUD and GD share common symptomatology and demonstrate common underlying genetic vulnerabilities [11], from which impulsivity is considered to be an endophenotypic indicator in both disorders [12,13]. Moreover, there is evidence that higher impulsivity may not only be present for the clinical presentation of GD, but may also be present in case of milder, subclinical problem gambling [3,14,15].

Impulsivity can be defined as a complex, multidimensional construct that is frequently described as the repetitive execution of maladaptive behaviours resulting in potential negative consequences. Hence, impulsive actions can be regarded as unplanned and rapid reactions to external or internal stimuli with the aim of obtaining immediate gratification and/or pleasure [12,16]. Contemporary models of impulsivity highlight the importance of not only behavioural aspects of impulsiveness, but also underlying neuropsychological components [17–19].

Impulsivity show complex neurobiological underpinnings, where the neurobiological and behavioural relationships between AUD, GD and impulsivity have been well documented [20,21]. The neurotoxic effects of chronic administration of alcohol and prolonged gambling behaviour are known to cause neural loss in “top-down” control regions of the brain, in the prefrontal cortex, the orbitofrontal cortex, the superior frontal association cortex, the supraoptic and paraventricular nuclei of the hypothalamus and the cerebellum, which have critical role in response inhibition, affective processing and decision-making, thus in impulsivity [22–24]. A diagnostic feature of both AUD and GD is the inability to abstain from the addictive behaviour even when facing severe negative consequences [25–27]. Such a failure in controlling one’s actions indicates inadequate inhibitory control, in which patients are unable to suppress the undesired, maladaptive act of gambling or drinking behaviour. This impairment reflects on deteriorated inhibitory control and decision-making, which represent a form of impulsivity [27,28].

Early impulsivity is proven to be a predictor of heavy drinking and also gambling behaviour in later life [29–31]. Moreover, there is evidence that the acute administration of alcohol and gambling activity induce impulsivity in humans [27,32]. This directly leads to impulsive behaviour evolving into compulsion that is linked to the development of the chronic forms of AUD and GD [33], where impulsivity has been frequently connected to poor clinical outcomes [34,35] and is associated with negative concomitant features like increased relapse risk [36] or increased alcohol consumption and more severe gambling behaviour [37–39].

Extensive literature exists on either diagnosed AUD or GD and their relations to impulsivity compared with healthy control groups, but up to now, only few studies have examined and compared AUD and GD patients in the same study in terms of any facet of impulsivity. [40] compared the differences of cognitive impulsivity measured by 2 neuropsychological tasks of 21 non-treatment-seeking problem or pathological gamblers and 21 AUD outpatients with healthy controls. They found shared deficits in ventral prefrontal cortical functions, while in tasks loading on dorsolateral prefrontal cortical functions were only impaired in AUD patients, presumably as a consequence of long-term alcohol consumption. [41] examined the differences of 3 decision-making tasks in 48 GD and 46 AUD outpatients and found that AUD patients performed marginally worse than the GD group. [42] studied the differences of a self-reported and a cognitive impulsivity task of 75 AUD and 44 GD patients recruited from mixed inpatient and outpatient settings. They revealed similar patterns of impulsivity in AUD and GD patients. These studies examined single diagnosis of AUD or GD; however, we could not identify studies that examined the subclinical emergence of problem gambling in hospitalised chronic AUD patients and its relations to impulsivity utilizing a comprehensive assessment battery.

Since impulsivity is not only proven to be a diagnostic criterion and a risk factor for AUD and GD, but the long-term alcohol exposure and gambling behaviour might further result in the impairment of impulse control, which prompts the emergence of AUD and GD. Moreover, a recently published meta-analysis indicated that the impairment of some executive functions, particularly impulsive decision-making were even higher in patients with a non-substance-related addictive disorder (GD) than in patients with the substance-related condition of AUD [43]. Based on these, it is paramount to explore the different aspects of impulsivity and their presentation in long-term AUD patients, and whether the existence of comorbid GD symptoms differentiate them in terms of impulsivity.

Due to the multidimensional nature of impulsivity, we aimed to explore it from a complex point of view, incorporating both objective and subjective measures of impulsiveness: i) traditional trait concept of impulsivity, which represents the execution of nonplanned actions and the engagement in maladaptive behaviours with disregarding potential future consequences [44,45]; ii) the inability of delaying gratifications or discarding rewards as a measure of choice impulsivity [46]; iii) impulsive aggression [47]; iv) deficits in response inhibition and decision-making [48,49].

To our knowledge, this is the first study that explores the associations of different aspects of impulsivity in chronic AUD patients compared to chronic AUD patients with comorbid GD symptoms. We hypothesised that those AUD patients who exhibit comorbid GD symptoms are distinct from AUD patients without GD symptoms by expressing higher symptom severity of substance use and demonstrating higher levels of trait impulsivity, choice impulsivity, impulsive aggression and impulsive decision-making.

## Methods

### Procedure

**Fig 1:**
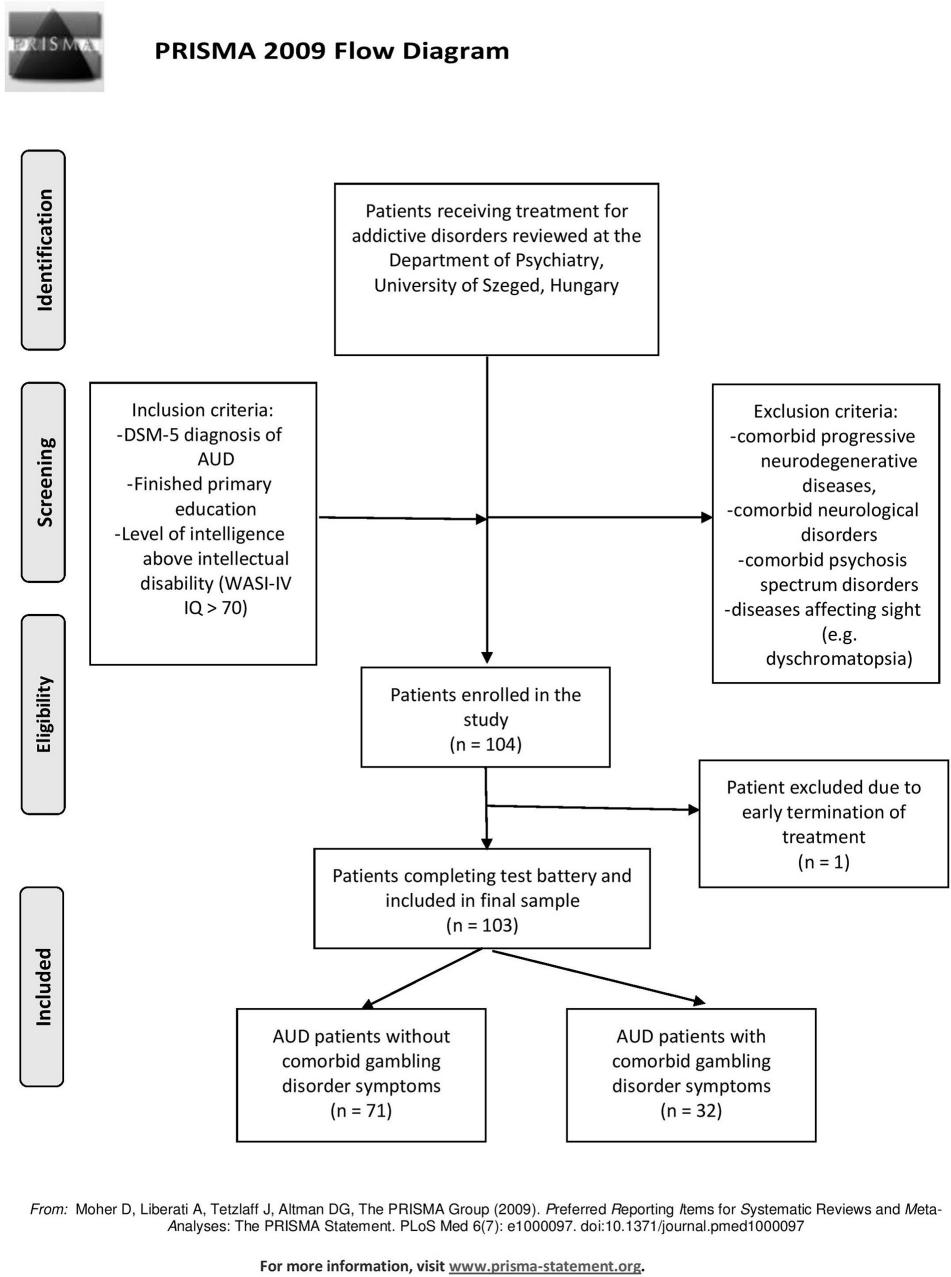
PRISMA flowchart of selection procedure. DSM: The Diagnostic and Statistical Manual of Mental Disorders, Fifth Edition; AUD: alcohol use disorder; WAIS-IV: The Wechsler Adult Intelligence Scale, Fourth Edition.

A comprehensive research project was conducted at the Department of Psychiatry, Faculty of Medicine, University of Szeged, Hungary. Patients receiving inpatient treatment for chronic alcohol use disorder (AUD) were assessed for executive functions, personality traits, addiction characteristics, comorbid psychiatric conditions, addictive disorders and psychopathological symptom severity. In this part of the study, which focuses on the evaluation of objective and subjective measures of impulsivity, a total of 104 patients were enrolled. Patients who met the inclusion criteria of having an established DSM-5 diagnosis of AUD, who finished at least primary education and whose level of intelligence surpassed the level of intellectual disability (Fourth Edition of the Weschler Adult Intelligence Scale above 70) were included. Patients who had a history of any psychosis spectrum disorder, progressive neurodegenerative disorders, neurological diseases, diseases affecting their sight or reported acute alcohol abuse were excluded from this study. One recruited patient was excluded due to voluntary termination of inpatient treatment; thus, the final sample size was 103. Patients were classified into two groups based on the presence of comorbid GD symptoms (see Fig 1).

The study was conducted in accordance with the Declaration of Helsinki and was approved by the Human Investigation Review Board, University of Szeged (ethical approval number: 49/B-53/2016KK). Written informed consent was obtained from each participant.

### Measures

#### Barratt Impulsivity Scale (BIS)

The Hungarian version of the BIS-11 is based on the original English version [45], which contains 30 items measuring three dimensions of impulsivity: motor impulsivity, attentional impulsivity and nonplanning. Items are measured on a scale ranging from 0 to 3; higher scores indicate higher impulsivity. The Hungarian version adapted by [50] showed good reliability in our sample: Cronbach’s α = 0.805.

#### Buss-Perry Aggression Questionnaire (BPAQ)

The Buss-Perry Aggression Questionnaire is a widely-used self-evaluation test for measuring aggressive impulses in 4 facets: physical and verbal aggression, hostility and anger [51]. The Hungarian version was adapted by [52], and showed good internal consistency and reliability (in our sample: Cronbach’s α = 0.879).

#### Delay Discounting Task (DDT)

In the Delay Discounting Task participants are presented with a series of monetary choices in which they have to decide between two different amounts of money, which they hypothetically receive in different time intervals. Once displayed, the rewards vary between 0HUF and 55,000HUF in 2,500HUF increments (1 EUR is about 320 HUF). The two choices differ in receival times, which vary in 0, 1, 14, 60, 180 or 365 days; the receival of one amount being closer in time (e.g. “now”) and the other being later (“in 2 weeks”). The task consists of 138 hypothetical questions that are presented in random order (i.e., Which of the two choices would you select? 10,000HUF now or 55,000HUF two weeks later?). Patients indicate their choices by a single click on their preferred selection, then they could continue the test by clicking on the ‘Next question’ button. The aim of the DDT is to determine the point at which the selection of the immediate reward was preferred over the delayed reward, which is known as the ‘indifference point’, and it can be computed for each period of delay. The participant selects the immediate available selection above this indifference point (which is given in HUF), while below the indifference point the participant chooses the option with delay.

#### Iowa Gambling Task (IGT)

The Iowa Gambling Task is one of the most frequently used and ecologically valid assessment tools for measuring decision-making, in which participants are asked to choose 100 times from four decks of cards with different win/loss ratio to win as much money as they can. For this, participants have to abandon short-term advantageous choices that would result in immediate high rewards accompanied with more/higher losses and instead, they need to select from decks, which result in lower immediate rewards, but lower long-term losses as well, turning out to be long-term advantageous choices [26,53]. For data analysis, choices were divided into five blocks (20 selections in each block). The amount of money won, and the total net score calculated by subtracting the number of advantageous selections from disadvantageous choices were also compared.

#### Wisconsin Card Sorting Task (WCST)

The WCST measures cognitive flexibility, executive functions and decision-making [54]. In this neuropsychological task, participants need to sort cards according to various criteria with the only feedback of the classification being correct or incorrect. Four sample cards are presented differing in pattern, colour and number of patterns to which participants need to match newly appearing cards. Cards can be classified according to their colour, their patterns or the number of patterns on each card. The classification rule changes every ten cards, which implies that when the participant has figured out the classification rule, they would eventually start making mistakes when the rule changes, and consequently they need to adapt to the changing rules. In our study we used the PEBL’s computerised version of card sorting task [55,56]. For data analysis, the number of correct responses, the number of incorrect responses and the number of perseverative errors were calculated.

#### Wechsler Adult Intelligence Scale (WAIS-IV)

The WAIS-IV is the most widely used intelligence scale to measure cognitive ability. The test consists of 10 subtests and 5 supplementary tests, which measure 4 major components of intelligence: Verbal Comprehension Index (VCI), Perceptual Reasoning Index (PRI), Working Memory Index (WMI) and Processing Speed Index (PSI) [57]. The Hungarian standardization was made by [58]. In this study, we calculated the WAIS-IV total score, which is the combined performance of the VCI, PRI, WMI and PSI.

#### Addiction Severity Index (ASI)

The ASI is a semi-structured interview, which covers 7 potential problematic areas (Medical, Employment/Support Status, Alcohol, Drug, Legal, Family/Social and Psychiatric) covering recent and lifetime substance-related problems [59]. The Hungarian adaptation was made by Rácz, Pogány & Máthé-Árvay (2002) [60]. From the interview, the following variables were selected: start of alcohol misuse in years, lifetime alcohol consumption in years, and abstinence during last 30 days.

#### South Oaks Gambling Screen (SOGS)

The South Oaks Gambling Screen is a 20-item questionnaire based on the DSM-III criteria of pathological gambling [61]. The Hungarian version was adapted by [62] and demonstrated good internal consistency. Scores between 1 and 4 show problematic gambling, while 5 or more points indicate probable pathological gambling.

#### Alcohol Use Disorders Identification Test (AUDIT)

The AUDIT is a 10-item self-evaluation screening test for assessing the severity of alcohol use and its adverse consequences developed by the World Health Organization [63]. The Hungarian adaptation by Gerevich, Bácskai & Rózsa (2006) [64] exhibited good validity and reliability (in our sample: Cronbach’s α = 0.763).

#### Symptom Checklist-90-R

The Symptom Checklist-90-R is a 90-item self-report questionnaire for assessing a broad range of currently existing psychopathological symptoms [65]. Items are rated on a 0–4 scale where more points indicate the presence of more severe symptoms during the past week. The test measures nine symptom dimensions, and one of its three global indices, the Global Severity Index (GSI) can be used as an indicator of the severity of psychopathological symptoms. The Hungarian version showed excellent internal consistency and reliability in our sample: Cronbach’s α = 0.953 [66,67].

#### Data analysis

Data analysis was conducted with IBM SPSS Statistics 24.0 software [68]. Based on the results of the SOGS, we divided our sample into two groups: 1) AUD who scored 0 on the SOGS were categorised as AUD patients without gambling symptoms (AUD group) and 2) those patients who scored 1 or above were labelled as AUD patients with probable or problem gambling symptoms (AUD+Gambling group). The DDT value was computed in Microsoft Excel 2016 with a hyperbolic equation fitted for each participant’s indifference point [69,70] with the use of the Solver subroutine:

$$V=\frac{Std}{1+kX}$$

where *V* stands for the value of the indifference point, *Std* stands for the amount of money available (55,000HUF), *k* is a fitted parameter which indexes the rate of discounting, and *X* represents the length of delay. The steepness of the curve (*k*) is fitted to the subjective value of each point of delay. When the curve is steeper (meaning that the *k* value is closer to zero), the individual prefers immediate rewards over delayed ones, which represents more impulsive choices.

Independent-samples *t*-tests were used for determining the group differences for continuous variables and Chi-square test was used for categorical variables to compare demographic parameters. Partial correlation analysis was used to reveal the relationship between gambling symptom severity and different facets of impulsivity. Comparison of the two groups’ performance on the IGT was determined by Repeated Measures ANOVA and Repeated Measures ANCOVA; due to the results of the Mauchly's Test of Sphericity, the Greenhouse-Geisser correction was applied. To examine the effect of demographic variables, psychopathology symptoms and measures of impulsivity on the likelihood that patients have problem gambling symptoms, binary logistic regressions were used with forward stepwise regression method [71].

Effect sizes were calculated using Cohen’s *d* [72], which is defined as the standardized difference between two means. According to [73], an effect size of 0.2–0.3 is considered to be a “small” effect size, 0.5 is a “medium” effect and above 0.8 is a “large” effect size.

## Results

### Sample characteristics

More than two-third (76%, n = 79 out of 103) of the participants were male. The mean age of participants was 45.7 years (SD = 10.35; age: 21–69). In total, 78.7% of the sample completed secondary education and 21.4% completed graduate education. Close to one third (31.1%) of the sample (n = 32) scored 1 or more points on the SOGS with scores ranging between 1 and 14 points (mean = 4.69, SD = 3.5); 18 people categorised as problematic gamblers and 14 as probable pathological gamblers. The two groups did not differ in gender, age, education, IQ, start of alcohol consumption or abstinence during the last 30 days, but the AUD+Gambling group was characterised by more severe alcohol use and longer lifetime alcohol consumption (see Table 1).

**Table 1:** Demographic, alcohol and gambling related characteristics of the sample.

	AUD (n = 71)	AUD+Gambling (n = 32)	
Gender *(M%)*	73.2%	84.3%	*Χ*^2^(1) = 1.530, *p* = 0.216)^a^
Education% (primary/secondary/higher)	5.6%/70.4%/24%	12.5%/71.9%/15.6%	*Χ*^2^(2) = 2.060, *p* = 0.357)^a^
Age (*SD*)	45.41(*9*.*612*)	46.28(*11*.*967*)	*t*(101) = -0.394, *p* = 0.694^b^
Start of alcohol misuse in years (*SD*)	25.50(*9*.*337*)	22.34(*11*.*449*)	*t*(100) = 1.473, *p* = 0.144 ^b^
Lifetime alcohol consumption in years (*SD*)	16.88(*9*.*856*)	21.63(*11*.*935*)	***t*(100) = -2.109, *p* = 0.037** ^b^
Abstinence duration during last 30 days (*SD*)	22.134(*15*.*571*)	17.594(*9*.*641*)	*t*(101) = 0.521, *p* = 0.131 ^b^
WAIS-IV Ttl IQ (*SD*)	92.32(*14*.*78*)	89.75(*15*.*917*)	*t*(101) = 0.798, *p* = 0.427 ^b^
AUDIT Total (*SD*)	23.62(*7*.*316*)	27.48(*6*.*961*)	***t*(100) = -2.489, *p* = 0.014** ^b^
SCL-90-R GSI (*SD*)	0.083(*0*.*064*)	0.110(*0*.*074*)	*t*(101) = -1.856, *p* = 0.066 ^b^

AUD: Alcohol use disorder patient group, AUD+Gambling: Alcohol use disorder patient group with problem or pathological gambling symptoms, WAIS Ttl IQ: Wechsler Adult Intelligence Scale IV total score

^a^ Chi-square test

^b^Independent sample *t*-test

### Exploratory correlation matrix and group differences between gambling symptom severity and demographic variables, psychopathology symptoms and measures of impulsivity

Partial correlation with age, lifetime alcohol consumption and SCL-90-R GSI as covariates were conducted to explore the associations between these variables, where the severity of gambling symptoms (SOGS scores) showed significant correlation with the BIS Total Score (r = 0.278, *p* = 0.006), while Impulsive aggression measured with the BPAQ Total score (r = 0.128, *p* = 0.209), and neuropsychological measures of impulsivity, as the number of correct responses in the WCST (r = -0.046, *p* = 0.658), the number of total errors in the WCST (r = 0.054, *p* = 0.602), the number of perseverative errors in the WCST (r = -0.068, *p* = 0.510), the DDT (r = -0.118, *p* = 0.254), the total win on the IGT (r = -0.005, *p* = 0.962), the number of advantageous choices on the IGT (r = 0.065, *p* = 0.529), the number of disadvantageous choices on the IGT (r = -0.065, *p* = 0.529) and the IGT net score (r = 0.065, *p* = 0.529) did not show significant connection with the severity of gambling symptoms (see S1 Annex).

**Fig 2:**
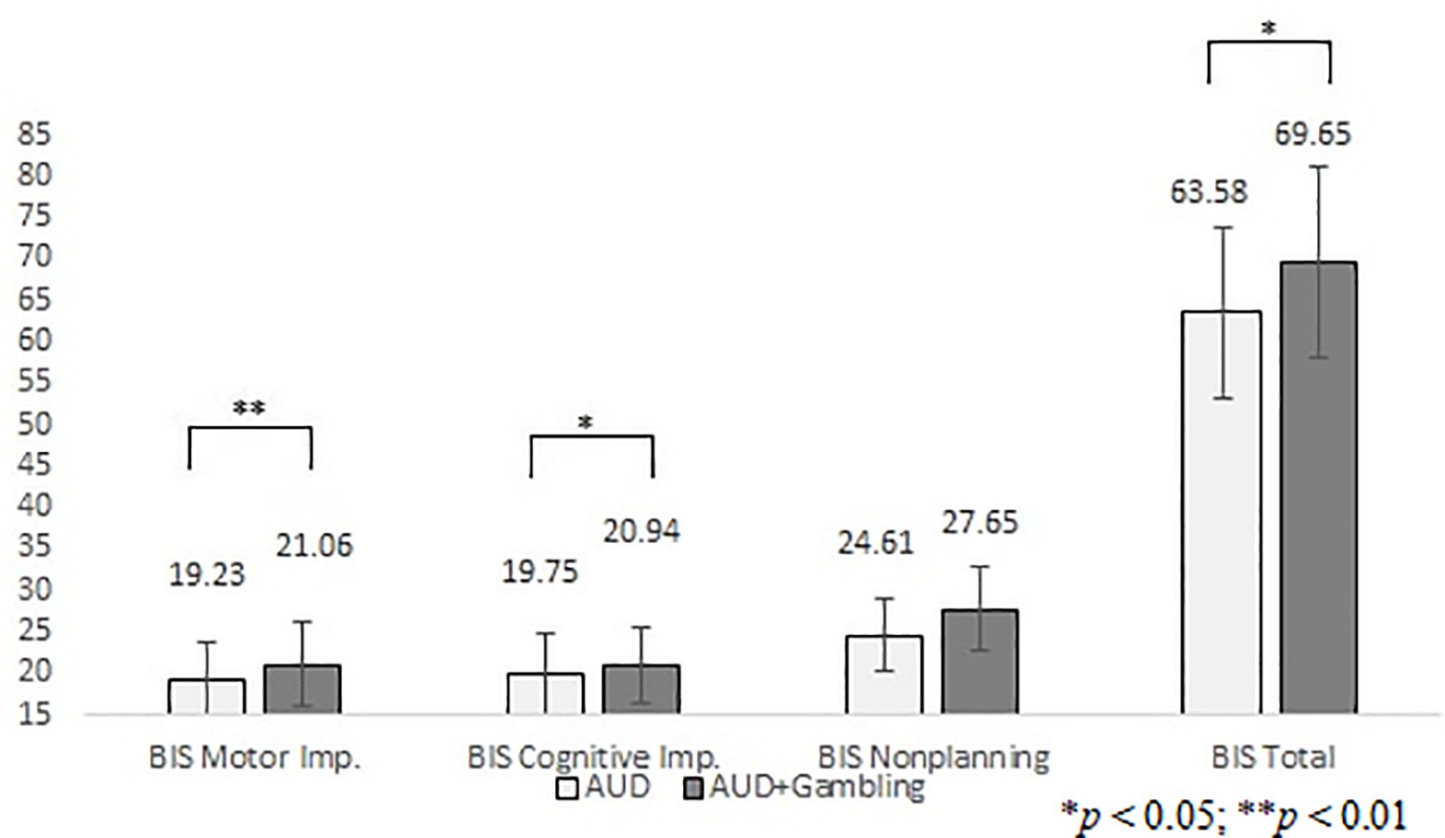
Group differences in Barratt Impulsivity Scale. AUD: chronic alcohol use disorder patients; AUD+Gambling: alcohol use disorder patients with problem or pathological gambling symptoms; BIS Motor Imp.: Barratt Impulsivity Scale Motor Impulsivity subscale; BIS Cognitive Imp.: Barratt Impulsivity Scale Cognitive Impulsivity subscale; BIS Nonplanning: Barratt Impulsivity Scale Nonplanning subscale; BIS Total: Barratt Impulsivity Scale Total score; the columns represent mean values, the error bars standard deviation.

Based on the associations explored in the correlation matrix, independent sample *t*-tests were conducted to explore group differences in the subscales of the BIS. Fig 2 illustrates that the AUD+Gambling group had higher scores in the BIS Nonplanning (*t*(100) = -3.024, *p* = 0.003, Cohen’s *d* = -0.634) and the BIS Total scores (*t*(100) = -2.635, *p* = 0.010, Cohen’s *d* = -0.555), and a tendency toward significance in the BIS Motor Impulsivity (*t*(100) = -1.767, *p* = 0.080, Cohen’s *d* = -0.371).

### The effect of demographic variables, psychopathology symptoms and measures of impulsivity on gambling symptoms

To explore the effect of demographic variables, psychopathology symptoms and measures of impulsivity on gambling symptoms, we performed two binary logistic regressions with forward stepwise regression method with AUD vs. AUD+Gambling as dependent variable. The first binary logistic regression was performed with age, gender, IQ measured by the WAIS-IV and SCL-90-R GSI as covariates. The BPAQ Total score, the BIS Total score, the number of correct responses in the WCST, the number of total errors in the WCST, the number of perseverative errors in the WCST, the DDT, the total win on the IGT, the number of advantageous choices on the IGT, the number of disadvantageous choices on the IGT and the IGT net score on the likelihood that patients have problem gambling symptoms were entered as predictors. Assumption of collinearity was tested and resulted in no indication of multicollinearity (Tolerance below 0.865 and VIF below 3.645 for every variable in the model). The baseline model (B = -0.895, S.E. = 0.220, Wald χ2(1) = 16.507, *p* ≤ 0.001, OR = 0.408) had an accuracy of 71.0% overall percentage. The binary logistic regression model was statistically significant (χ2(1) = 7.324, *p* = 0.007; R^2^ = 0.101; Hosmer-Lemeshow goodness-of-fit test: χ2(8) = 2.703, *p* = 0.958). Increasing BIS Total score was associated with the increased likelihood of having problem gambling symptoms (B = 0.057, S.E. = 0.022, Wald χ2(1) = 6.631, *p* = 0.010, OR = 1,059, 95% CI = 1.014–1.105) while all the other variables had a non-significant effect in the final model.

In the second binary logistic regression the BIS and BPAQ subscales were also included besides the total scores, namely: BIS Motor Impulsivity Score, BIS Cognitive Impulsivity Score, BIS Nonplanning Score, BPAQ Verbal Aggression Score, BPAQ Physical Aggression, BPAQ Hostility score and BPAQ Anger score. Additionally, the number of correct responses in the WCST, the number of total errors in the WCST, the number of perseverative errors in the WCST, the DDT, the total win on the IGT, the number of advantageous choices on the IGT, the number of disadvantageous choices on the IGT and the IGT net score were included as predictors with age, gender, IQ measured by the WAIS-IV and SCL-90-R GSI as covariates. Assumption of collinearity was tested and resulted in no indication of multicollinearity (Tolerance below 0.819 and VIF below 3.808 for every variable in the model). The baseline model (B = -0.895, S.E. = 0.220, Wald χ2(1) = 16.507, *p* ≤ 0.001, OR = 0.408) had an accuracy of 70.0% overall percentage. The binary logistic regression model was statistically significant (χ2(1) = 8.914, *p* = 0.003; R^2^ = 0.122; Hosmer-Lemeshow goodness-of-fit test: χ2(7) = 9.121, *p* = 0.244). Increasing BIS Nonplanning score was associated with the increased likelihood of having problem gambling symptoms (B = 0.143, S.E. = 0.051, Wald χ2(1) = 7.844, *p* = 0.005, OR = 1,154, 95% CI = 1.044–1.275), while all the other variables had a non-significant effect in the final model.

## Discussion

Hospitalized patients with long-term chronic alcohol use disorder (AUD) with or without gambling disorder (GD) symptoms were compared on an extensive test battery that assessed four sub-dimensions of impulsivity. The traditional trait concept of impulsivity was measured by the Barratt Impulsivity Scale (BIS), which is the most commonly used internally consistent measure of trait impulsivity in clinical setting. The ability of postponing gratifications or delaying immediate rewards was assessed by the Delay Discounting Task (DDT), which is a widely accepted method for understanding impulsive choices. Impulsive aggression was examined with the Buss-Perry Aggression Questionnaire (BPAQ), and deficits of decision-making and response inhibition, as measurements of cognitive impulsivity, were assessed with the Iowa Gambling Task (IGT) and the Wisconsin Card Sorting Task (WCST).

In our sample, the concurrent AUD and problem or pathological gambling symptoms were associated with more severe alcohol use, longer lifetime alcohol consumption and higher levels of trait impulsivity measured by the BIS. When controlled for age, lifetime alcohol consumption and SCL-90-R GSI, GD symptom severity showed significant correlation with trait impulsivity, and only trait impulsivity was related to the increased likelihood of having GD symptoms. Overall, these findings were not due to the result of group differences in age, nor were they mediated by the measured differences in years of alcohol consumption or psychiatric symptom severity evaluated in this study.

Our results indicate that only higher trait impulsivity was associated with comorbid GD symptoms in chronic AUD patients, which was independent of intelligence, age, gender and psychopathological symptom severity. Moreover, the nonplanning aspect of trait impulsivity was associated with the occurrence of comorbid GD symptoms in chronic AUD. This link conforms to previous literature, since in functional imaging studies, the nonplanning dimension of impulsivity measured with the BIS correlated with volumes of the right middle cingulate gyrus, the left anterior cingulate gyrus, left middle frontal gyrus, left middle cingulate gyrus and right orbitofrontal gyrus [74]. Frontal lobe dysfunction is a leading symptom of the alcohol-related impairment in the brain that prolonged AUD causes [75], thus the risk of developing comorbid GD in chronic AUD is especially high.

In our sample of long-term chronic AUD patients, trait impulsivity proved to be a determinant factor, in which AUD patients with comorbid GD symptoms exhibited higher trait impulsivity than patients without them, and trait impulsivity was also associated with more severe substance use symptoms. There is mount evidence on the negative consequences of trait impulsivity in alcohol use and gambling as well, expressed both in clinical and subclinical forms. [76] examined the role of trait impulsivity measured with the BIS in excessive alcohol consumption and alcohol misuse and proved the role of trait impulsivity as a risk factor in alcohol misuse. Concerning diagnosed AUD, several epidemiological [77], cross-sectional [36,41] and longitudinal studies [78,79] thoroughly supported the maladaptive role of higher trait impulsivity in AUD patients. Similarly in GD, [80] compared non-problematic, at-risk and problem gamblers in terms of cognitive impulsivity and found that at-risk and problem gamblers also showed elevated BIS Motor and Attentional Impulsivity. Also in case of clinically diagnosed GD, several studies verified higher trait impulsivity compared to healthy controls [81,82].

Impulsive choice-making is a predominant feature both in AUD and GD. [83] showed a more rapid discounting of delayed rewards among AUD patients, while [25] indicated that GD symptom severity is associated with higher choice impulsivity in a delay discounting task. [84] examined substance abusers with and without problem gambling and indicated that substance abusers with GD symptoms discounted delayed rewards more rapidly than their patients without GD symptoms. Even though it has been previously verified that GD had an additive effect on delay discounting rates, in our study delay discounting was not associated with GD symptoms nor did it increase the likelihood of having GD symptoms in AUD patients. A reason for that may lie in the fact that patients suffering from addictive disorders with or without GD symptoms are documented to discount delayed rewards more rapidly than healthy controls, thus addictive disorders themselves are associated with higher discounting rates [85,86].

Previous studies have explored that poor response inhibition—that may be related to impulsive aggression—is affected by acute alcohol consumption [87–89] and chronic alcohol abuse and dependence as well [90,91]. The control of response inhibition is mediated by prefrontal/orbito-frontal and limbic/thalamic cortical circuits, and the impairment of these interconnected circuitries may induce excessive aggressive responding, thus impulsive aggression [92,93]. In our study, we measured impulsive aggression with the BPAQ, which is a widely used self-evaluation test in clinical setting for assessing trait aggression. In our sample, BPAQ total scores correlated with gambling symptom severity, which conform to current scientific results. Similarly, growing number of studies have reported a link between gambling and aggressive behaviour [94]. While in a longitudinal study of males tested at age 12, 15 and 18, [95] found that early aggressive behaviour measured with the BPAQ leads to the increase of alcohol consumption, but they did not find that alcohol use have led to later aggressive behaviour. Similarly, the presence of aggressive impulses has been well-documented in clinical populations of AUD and GD as well [96,97].

Risky decision-making and response inhibition as forms of impulsivity have been hypothesised to play a central role in addictive processes. Gambling tasks with risks and rewards like the IGT and tasks measuring executive (frontal lobe) functions like the WCST have been widely used to assess decision-making capacities and deficits in response inhibition in individuals with AUD and GD. Compared to healthy controls, AUD and GD patients both exhibit deficits on the IGT, meaning that they more frequently choose the larger immediate reward despite the presence of a larger concomitant punishment [98–101]. Similarly, it has also been documented that in case of chronic long-term alcohol consumption the performance on executive function tests like the WCST deteriorated [102,103]; also a previous study indicated that GD patients had more perseverative errors on the WCST, which is another measure of cognitive flexibility [104]. Our results do not conform to these previous findings, since in our sample we could not identify differences on the IGT or the WCST between chronic AUD patients with or without GD symptoms, nor did these test results contribute to the likelihood of having GD symptoms. The reason behind the lack of difference between long-term AUD and AUD with comorbid GD symptoms might be reasoned with the effects of chronic alcohol consumption in those cortical regions that play essential role in response inhibition and decision-making [41,105,106], which in this case did not result in the even higher deficit of the neurocognitive performance of AUD patients with symptoms of GD compared to patients with only AUD.

Regarding the lack of differences in other aspects of impulsivity measured in this study, meta-analyses [18,19] indicate that impulsivity is not a unitary construct and it has different manifestations in AUD and GD compared to healthy populations. Ioannidis et al. (2019) argued that clinical and even subclinical GD is characterized by general disfunction in inhibitory control, thus impulsive cognitive disfunction, while another meta-analysis [71] established that both AUD and GD show impairment in cognitive impulsivity, GD patients exhibiting even higher impairment on impulsive decision-making.

However, a few limitations need to be taken into account when interpreting the results of this study. Since impulsivity is regarded as a complex and multifactorial phenomenon, there is no clear-cut consensus for defining and operationalizing each component of it, even the number of components and their separability are questioned. We operationalized impulsivity by neuropsychological and self-measurement tests assessing four domains: trait impulsivity, choice impulsivity, impulsive aggression and impulsive decision-making; thus, the present study is limited by this notion.

Additionally, in the present study, impulsive aggression was measured by the Buss-Perry Aggression Questionnaire (BPAQ), which is related to impulsiveness; however, it does not explicitly measure impulsivity, since it includes items of premeditated antisocial behaviour and hostility. Moreover, considering the cross-sectional nature of the current study, conclusions concerning causality between comorbid AUD and GD symptoms and distinct facets of impulsivity cannot be drawn. In this study, we could only establish a link between longer lifetime alcohol consumption, more severe alcohol consumption and heightened trait impulsivity in AUD patients with comorbid GD symptoms compared to sole AUD. Since the prolonged misuse of alcohol leads to impulsive actions across one's life course, and this influence both objective and subjective measures of impulsivity. Future research would benefit from the longitudinal evaluation of the different aspects of impulsivity in chronic AUD populations. Taking into consideration its different presentations in AUD comorbid with GD symptoms may contribute to clearing directions for providing target-specific and effective treatment approaches.

Supplementary information for this manuscript can be found online: https://dx.doi.org/10.6084/m9.figshare.11473296

## Supporting information

S1 Annex Correlation matrix for study variables. (DOCX)
