# Supplementary material for: Different aspects of impulsivity in chronic alcohol use disorder with and without comorbid problem gambling
Source: PLoS One. 2020 Jan 30;15(1):e0227645. doi: 10.1371/journal.pone.0227645 (PMC6992191; doi:10.1371/journal.pone.0227645)
Supplement: S1 Annex — (DOCX) [file pone.0227645.s001.docx]

Annex 1: correlation matrix for study variables

|  | SOGS  Total | Gender | WAIS  Ttl. IQ | BIS  Total | BPAQ  Total | WCST  correct | WCST  incorrect | WCST  pers. err. | DDT | IGT  total win | IGT  adv. ch. | IGT  disadv. ch. | IGT  net score |
| --- | --- | --- | --- | --- | --- | --- | --- | --- | --- | --- | --- | --- | --- |
| SOGS Total | 1 |  |  |  |  |  |  |  |  |  |  |  |  |
| Gender | -0.121 | 1 |  |  |  |  |  |  |  |  |  |  |  |
| WAIS Ttl. IQ | -0.089 | 0.008 | 1 |  |  |  |  |  |  |  |  |  |  |
| BIS Total | **0.278*** | -0.051 | -0.168 | 1 |  |  |  |  |  |  |  |  |  |
| BPAQ Total | 0.134 | -0.039 | -0.118 | 0.158 | 1 |  |  |  |  |  |  |  |  |
| WCST correct | -0.046 | -0.037 | **0.522*** | **-0.222*** | -0.055 | 1 |  |  |  |  |  |  |  |
| WCST incorrect | 0.054 | 0.041 | **-0.518*** | **0.213*** | 0.047 | **-0.997*** | 1 |  |  |  |  |  |  |
| WCST pers. err. | -0.068 | -0.158 | **-0.248*** | 0.161 | 0.080 | -0.170 | 0.168 | 1 |  |  |  |  |  |
| DDT | -0.118 | 0.164 | -0.008 | -0.015 | -0.110 | -0.037 | 0.037 | -0.069 | 1 |  |  |  |  |
| IGT total win | -0.005 | **-0.253*** | 0.170 | -0.198 | -0.076 | 0.184 | -0.191 | -0.091 | -0.118 | 1 |  |  |  |
| IGT adv. ch. | 0.065 | -0.160 | **0.305*** | -0.161 | -0.101 | **0.290*** | **-0.287*** | -0.101 | -0.083 | **0.810*** | 1 |  |  |
| IGT  disadv. ch. | -0.065 | 0.160 | **-0.305*** | **0.161** | 0.101 | **-0.290*** | **0.287*** | 0.101 | 0.083 | **-0.810*** | **-1*** | 1 |  |
| IGT net score | 0.065 | -0.160 | **0.305*** | -0.161 | -0.101 | **0.290*** | **-0.287*** | -0.101 | -0.083 | **0.810*** | **1*** | **-1*** | 1 |

*correlation is significant at the 0.05 level

SOGS Total: South Oaks Gambling Scale total score; WAIS Ttl IQ: intelligence score measured with WAIS-IV; BIS Total: Barratt Impulsivity Scale total score; BPAQ Total: Buss-Perry Aggression Questionnaire total score; WCST correct: Wisconsin Card Sorting Task correct answers; WCST incorrect: Wisconsin Card Sorting Task incorrect answers; WCST pers. err.: Wisconsin Card Sorting Task perseverative errors; DDT: Delay Discounting Task; IGT total win: Iowa Gambling Task total amount of money won; IGT adv. ch.: Iowa Gambling Task total number of advantageous choices; IGT disadv. ch.: Iowa Gambling Task total number of disadvantageous choices; IGT net score: Iowa Gambling Task net score
